# Human Data on Bisphenol A and Neurodevelopment

**DOI:** 10.1289/ehp.0901610

**Published:** 2009-12

**Authors:** Matthew P. Longnecker

**Affiliations:** Epidemiology Branch, National Institute of Environmental Health Sciences, National Institutes of Health, Department of Health and Human Services, Research Triangle Park, North Carolina, E-mail: longnec1@niehs.nih.gov

In this issue of *EHP*, Braun et al. (2009) report that the concentration of bisphenol A (BPA) in maternal urine from early pregnancy is associated with female offspring having more externalizing behavior. Hyperactivity and aggression are externalizing behaviors, and both are more frequent in boys than in girls (Hölling et al. 2008; Stene-Larsen et al. 2009). Sexually dimorphic behaviors in female rodents have been shown to be masculinized by exogenous estrogens (Ryan and Vandenbergh 2006), and BPA is weakly estrogenic in most experimental systems (Kuiper et al. 1998). Early pregnancy is the time in humans when masculinizing hormones first have their effects on the human brain (Cohen-Bendahan et al. 2005). I congratulate Braun and colleagues for bringing forth epidemiologic data on a topic for which it is most welcome and timely. Regulators at the U.S. Food and Drug Administration are currently reconsidering policy on BPA (U.S. FDA 2009). Thus, interpretation of the new results needs especially careful consideration.

Although the conclusions reached by Braun et al. (2009) may appear to be supported by the experimental literature, the role of estrogen in development—especially in the brain—is different in rodents and primates (Witorsch 2002). Although plasma estrogens increase in both rodents and primates during pregnancy, the increase in humans (Burney et al. 2008) is > 3 times that in rodents (González et al. 2003; Rodríguez-Cuenca et al. 2006); the absolute difference in estrogen levels between species is even greater (Witorsch 2002). More important, in the developing male rodent brain, testosterone is converted to estrogen, and it is this estrogen that is responsible for masculine behavior (Li et al. 2008). In rodents, a masculinizing effect of low-dose BPA has been demonstrated (Chapin et al. 2008; Ryan and Vandenbergh, 2006). In developing male primate brains, however, testosterone appears to masculinize directly without an estrogen intermediary (Li et al. 2008; Wallen and Hassett 2009). The synthetic estrogen diethylstilbestrol, when administered during human pregnancy, has no established effects on behavior of female offspring (Cohen-Bendahan et al. 2005). According to Cohen-Bendahan et al. (2005), “prenatal estrogen appears to have little effect on early human development, perhaps because both males and females are exposed to high levels of estrogen from the mother.” Furthermore, Li et al. (2008) stated that “to the extent that endocrine disrupters such as bisphenol A have been shown to duplicate or disrupt estradiol’s action in the developing rodent nervous system …, the relevance of such effects for human brain and behavioral development is called into question.”

BPA, however, could have effects on the developing human brain that result from interaction with the androgen receptor (Sun et al. 2006) or that are due to interference with effects of estrogens on neural circuitry or plasticity that are unrelated to sexual differentiation (Leranth et al. 2008). However, it is unclear whether such effects might occur at low BPA doses such as those to which humans are exposed. Thus, with respect to an assessment of a biologically plausible mechanism for Braun et al.’s findings of an association in human females only (Braun et al. 2009), the literature is ambiguous and not especially supportive.

Although the sexual dimorphism of externalizing behavior is widely recognized, absolute differences in externalized scores associated with BPA cannot be determined using the sex-standardized data presented (see Figure 1 in Braun et al. 2009). Thus, the size of the association with BPA in girls cannot be compared with the size of the male–female difference. Without this absolute difference (and corresponding sex-specific data on distributions) for comparison, we cannot know whether the largest estimated “effect” of BPA exposure is to produce girls who behave like boys or girls who still behave like girls. Such a close interpretation, before the results are confirmed by others, is perhaps premature.

The most important information provided by Braun et al. (2009) may be the correlation among urinary levels of BPA (on a creatinine basis) at different times during pregnancy (≤ 0.11). This means that measuring a single urine sample provides little information about longer-term exposure. This is believable because labeled BPA has a half-life of hours in humans (Völkel et al. 2002), and exposure appears to vary from day to day (Nepomnaschy et al. 2009). If this degree of difficulty in characterizing longer-term exposure in pregnancy is true in general of BPA, epidemiologists face a challenge in finding true associations between developmental exposure with outcomes, should any exist.

With the challenge in characterizing exposure now more clear, the exploration of other strategies may be a priority. For example, BPA can form adducts with DNA *in vivo* (Atkinson and Roy 1995; Izzotti et al. 2009). Could adducts of BPA with albumin be detectable and be a better biomarker of exposure in humans? Other, improved biomarkers of exposure in humans are also conceivable, although they may be less feasible in routine studies (Fernandez et al. 2007).

Vigilance regarding potential adverse effects of ubiquitous, low-level exposures is a necessity of modern life. Braun et al. (2009) present a complete analysis of data on a critical topic. This initial report, however, may raise unrealistic expectations about what epidemiologic studies can contribute on this topic. Their findings bring to mind Tufte’s Evidence Decay Cycle: “too often the first published study testing a new treatment provides the strongest evidence that will ever be found” (Tufte 2006). Given the potential implications of Braun et al.’s findings to human health, let us hope that these findings will not be confirmed in humans and that the best evidence of adverse effects of BPA will come from toxicology studies.

## Figures and Tables

**Figure:**
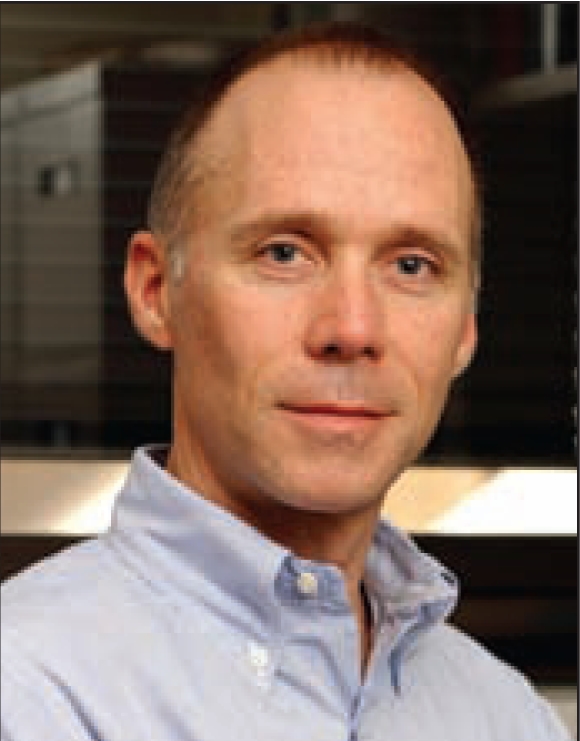
Matthew P. Longnecker
